# Qiliqiangxin capsule alleviates cardiac hypertrophy and cardiac dysfunction by regulating miR-382-5p/ATF3 axis

**DOI:** 10.1016/j.clinsp.2024.100496

**Published:** 2024-09-26

**Authors:** Bao Yin, XiaoTong Jiang, XinFeng Chang, ChunHua Song

**Affiliations:** a Department of Cardiovascular, Zibo Hospital of Traditional Chinese Medicine, Zibo City, Shandong Province, China; b Department of Human Anatomy, Jiangsu Vocational College of Medicine, Yancheng City, Jiangsu Province, China; c Department of Surgery, Jiangsu Vocational College of Medicine, Yancheng City, Jiangsu Province, China

**Keywords:** Qiliqiangxin capsule, miR-382-5p, ATF3, Cardiac hypertrophy, Cardiac dysfunction

## Abstract

**Objective:**

Qiliqiangxin Capsule (QL) was investigated for its possible role in cardiac hypertrophy in this study.

**Methods:**

QL (0.5 mg/mL) was pre-treated in Neonatal Mouse Ventricular Cardiomyocytes (NMVCs) before induction of cardiomyocyte hypertrophy by Angiotensin II (Ang-II). Immunofluorescence staining for α-actinin was conducted to determine cell surface area. Atrial Natriuretic Peptide (ANP) and Brain Natriuretic Peptide (BNP) of hypertrophy markers were examined. Ang-II infusion was given to stimulate cardiac hypertrophy in mice. The cardiac function of mice was detected by echocardiography, and the pathological status of myocardial tissue was observed.

**Results:**

The surface of cardiomyocytes was enlarged by Ang-II, and ANP and BNP levels were increased. QL processing could save these changes. miR-382-5p was upregulated in Ang-II-treated NMVCs, and reducing miR-382-5p could further enhance the therapeutic effect of QL while elevating miR-382-5p weakened the protective effect of QL. QL could inhibit miR-382-5p expression to negatively regulate Activated Transcription Factor 3 (ATF3) expression. Enhancing ATF3 expression rescued miR-382-5p upregulation-mediated role in NMVCs. In addition, QL alleviated Ang-II-stimulated cardiac hypertrophy and cardiac dysfunction in mice.

**Conclusion:**

QL may alleviate cardiac hypertrophy and cardiac dysfunction via the miR-382-5p/ATF3 axis.

## Highlights

• QL alleviates cardiomyocyte hypertrophy.
• Silencing miR-382-5p could further enhance the therapeutic effect of QL.
• miR-382-5p negatively regulates ATF3 expression.
• ATF3 upregulation can reduce the hypertrophic effect of upregulation of miR-382-5p in NMVCs.

## Introduction

Adaptive to different stimuli such as hypertension, ischemic heart disease, and cardiomyopathy, cardiomyocyte hypertrophy involves enlargement, apoptosis, and fetal gene activation. Cardiovascular hypertrophy, although initially compensatory, leads to cardiac dysfunction, heart failure, and even sudden death over time.^1^, ^2^, ^3^, ^4^

In 2004, Qiliqiangxin (QL), a traditional Chinese medicine comprising a combination of 11 distinct herbs including astragali radix, ginseng radix et rhizoma, semen descurainiae lepidii, aconiti lateralis radix preparata, among others, received approval for heart failure treatment in China.^5^ Patients with chronic heart failure treated with QL showed a greater decrease in N-Terminal (NT) pro-B-type Natriuretic Peptide (BNP) levels than those treated with standard treatment.^6^ Furthermore, QL has the potential to reinstate cardiac functionality, mitigate cardiac hypertrophy and failure, and enhance mitochondrial function.^7^, ^8^, ^9^ By activating the Peroxisome Proliferator-Activated Receptor-γ (PPAR-γ) and mTOR pathway, respectively, QL can promote cardiac remodeling, thereafter to alleviate Myocardial Infarction (MI) or ischemia-reperfusion injury.^10^^,^^11^ The administration of QL has the potential to induce an increase in mitochondrial biogenesis within cardiomyocytes, while simultaneously inhibiting the differentiation of cardiac fibroblasts,^12^^,^^13^ but QL does not appear to have a clear mechanism for its protective effects against cardiac hypertrophy.

A range of biological processes are mediated by small noncoding RNAs, and miRNAs including cell growth, differentiation, apoptosis,^14^ tissue injury, oxidative stress, and metabolism.^15^^,^^16^ MiR-382-5p may also be a biomarker for aortic stenosis in addition to liver and kidney injury.^17^^,^^18^ In addition, down-regulating miR-382-5p can reduce cardiomyocyte apoptosis after acute MI.^19^ It is suggested that miR-382-5p is a miRNA associated with heart diseases.

Limited knowledge exists regarding the regulatory mechanism of QL in the context of cardiac hypertrophy. Consequently, the objective of this investigation was to examine the association between QL and cardiac hypertrophy. The findings of this study provide evidence that QL mitigates cardiac hypertrophy through the miR-382-5p/Activated Transcription Factor 3 (ATF3) axis.

## Materials and methods

### Neonatal mouse ventricular cardiomyocytes (NMVCs) collection and treatment

Animal experiments in this study followed the ARRIVE guidelines. NMVCs were isolated from 1‒3-day-old neonatal C57BL6 mice. In short, the ventricles were dissected, cleaned, and chopped in 1× ADS buffered saline solution (200 mL 10× ADS: 9.52g HEPES, 13.6g NaCl, 1.2g glucose, 0.276g Na_2_HPO_4_, 0.0102g MgSO_4_, 0.8g KCl, 200 mL H_2_O, Ph 7.35∼7.45). The tissue was digested in 1× ADS buffered saline solution containing 0.4 mg/mL collagenase type II (Worthington, USA) and 0.6 mg/mL trypsin (Sigma, USA) at 37°C. NMVCs were centrifuged and re-suspended in high-glucose Dulbecco's modified Eagle's medium (Gibco, USA) containing 10% fetal bovine serum (Gibco), 5% horse serum (Hyclone, USA), 1% penicillin-streptomycin, and 0.1 mM 5′-Bromo-2′-Deoxyuridine (Sigma). The cells were then plated in different petri dishes coated with 10 mg/mL gelatin (Sigma) according to specific experimental requirements.

NMVCs were treated with 10‒8 M Angiotensin II (Ang-II) (MedChemExpress, NJ, USA) for 48h*,* while treated with or without 0.5 μmoL/mL QL for 48h (Shijiazhuang Yiling Pharmaceutical Co., Ltd., China). NMVCs pre-treated with QL were transfected with oligonucleotides or plasmid vectors (GeneChem, Shanghai, China) that interfered with miR-382-5p or ATF3 expression using Lipofectamine 2000 (Invitrogen, MA, USA). At 12h post-transfection, NMVCs were treated with Ang-II for 48h.

### Immunofluorescent staining

Following the washing with PBS, NMVCs were subsequently fixed with a 4% paraformaldehyde solution for 20 min. Subsequently, NMVCs were blocked with 10% goat serum for 1h. This was followed by an overnight culture with α-actinin (1:500, Sigma). Cy3-AffiniPure goat anti-mouse IgG (Jackson ImmunoResearch, USA), in combination with 4′,6-diamidino-2-phenylindole, was incubated with NMVCs after three washes with PBS. Using ImageJ Launcher software, the cell surface area was quantified using images taken with a Nikon Eclipse Ti microscope.

### Cardiac hypertrophy mouse model

Model group and Sham group were established with 9-week-old mice, 6 mice in each group. Cardiac hypertrophy was induced by Ang II infusion (MedChemExpress, NJ, USA) at 1000 ng/kg/min with osmotic micropump (Alzet Model 2004) for 3 consecutive weeks, and the Sham group was injected with the same dose of normal saline. The pump was initially filled with Ang II or normal saline and incubated in sterile normal saline at 37°C for 48h. The mice were subjected to anesthesia using a 3.0% isoflurane-O_2_ mixture. Following the administration of anesthesia, an incision was made on their backs, and subsequently, a pump was inserted into the subcutaneous nerve region. Finally, a suture was applied to close the incision. Postoperative analgesia was achieved by buprenorphine at 0.1 mg/kg. The mice that regained consciousness were put back in their cages and fed.

QL (n = 6), QL + AAV9-NC (n = 6), QL + AAV9-miR-382-5p mimic (n = 6), and QL + AAV9-si-ATF3 (n = 6) groups were constructed. Each mouse was given QL gavage (0.6 mg/kg/d) while the AAV-9 vector containing 2.5 × 10^11^ viral genome particles (GeneChem) was injected through the tail vein. After 4 weeks, Ang-II infusion was performed as indicated above to stimulate cardiac hypertrophy.

### Echocardiography and histopathological examination

Mice were anesthetized by inhalation of 1.0% isoflurane after Ang-II infusion and examined by echocardiography using the Ultrasound Imaging System (VisualSonics Vevo 3100) to assess their cardiac function. Left Ventricular end-diastolic Anterior Wall thickness (LVAWs), left ventricular Ejection Fraction (EF), and left ventricular shortening Fraction (FS) were recorded. Subsequently, the mice were euthanized to excise the broadest segment of the heart, which was subsequently immersed in 4% paraformaldehyde for an overnight fixation period and subsequently embedded in paraffin. The resulting sections, measuring 5 μm, were subjected to Hematoxylin and Eosin (HE) staining (Sigma). The sections were incubated with Wheat Germ Agglutinin (WGA, Sigma) to evaluate the cross-sectional areas of cardiomyocytes.

### RT-qPCR

Total RNA was extracted from cells or tissues using the miRNeasy Mini Kit (Tiangen, Beijing, China). Bio-Rad iScript^TM^ cDNA Synthesis Kit was used to synthesize cDNA. Atrial natriuretic peptide (ANP), ATF3, and BNP were detected by ABI-7900 PCR system using Tli RNaseH Plus (Takara, Tokyo, Japan) and normalized with glyceraldehyde-3-phosphate dehydrogenase (GAPDH). miR-382-5p was determined by Bulge Loop^TM^ miRNA qPCR Primer Set (RiboBio, Guangzhou, China) and Takara SYBR Premix Ex Taq^TM^ in the ABI-7900 real-time PCR assay system. U6 was an internal standardization reference. The primers are shown in Table 1. Each gene was calculated by 2^−ΔΔCt^.

**Table 1:** Primers used in RT-qPCR.

Genes	Primer sequences (5’–3’)
microRNA-382-5p	Forward: GAAGTTGTTCGTGGTGGATTCG
	Reverse: TATGGTTGTAGAGGACTCCTTGAC
ATF3	Forward: CGAAGACTGGAGCAAAATG
	Reverse: AGGTTAGCAAAATCCTCAAATAC
ANP	Forward: ACAACTGGTATTGTGCTGGACT
	Reverse: TCAGCAGTAGTCACGAAGGAAT
BNP	Forward: CAGAACAATCCACGATGCAG
	Reverse: GCCGATCCGGTCTATCTTCT
U6	Forward: CTCGCTTCGGCAGCACA
	Reverse: AACGCTTCACGAATTTGCGT
GAPDH	Forward: CATCAACGGGAAGCCCATC
	Reverse: CTCGTGGTTCACACCCATC

ATF3, Activated Transcription Factor 3; ANP, Atrial Natriuretic Peptide; BNP, B-Type Natriuretic Peptide; GAPDH, Glyceraldehyde-3-Phosphate Dehydrogenase.

### Western blot

The Whole-Cell Lysis Assay (KeyGEN BioTECH) was employed to extract total protein from cells and tissues, while the BCA protein assay kit (KeyGEN BioTECH) to determine protein concentration. The protein extract underwent boiling and denaturation, followed by separation using a 10% sodium dodecyl sulfate-polyacrylamide gel electrophoresis technique, and subsequently transferred onto a polyvinylidene difluoride membrane (Millipore). Following the blocking step with 5% skim milk powder, the membrane was probed with rabbit primary antibodies at 4°C for an overnight duration: ANP (1:1000, ab189921, Abcam), BNP (1:1000, ab239510, Abcam), ATF3 (1:1000, ab207434, Abcam), and GAPDH (1:1000, ab8245, Abcam). Afterward, the membrane was subjected to incubation with a secondary antibody conjugated with horseradish peroxidase at ambient temperature for 1h. The detection of the membrane was accomplished through enhanced chemiluminescence provided by KeyGEN BioTECH, in conjunction with a chemiluminescence system manufactured by Bio-Rad.

### Dual-luciferase reporter gene assay

A targeted binding site between miR-382-5p and ATF3 was identified by the bioinformation website starBase. psiCHECK2 plasmid (Promega) containing wild-type ATF3 (ATF3-WT) or mutant ATF3 (ATF3-MUT) was constructed at the speculated miR-382-5p binding site. Plasmid ATF3-WT and ATF3-MUT, harboring either miR-382-5p mimic or negative control, were transfected into HEK293T cells using Lipofectamine 2000 (Invitrogen) for 48h. The lysate of HEK293T cells was subsequently collected to assess luminescence levels (firefly luciferase as control) using the SpectraMax M5 (Molecular Devices).

### Statistical analysis

All data were analyzed using SPSS 22.0 software and expressed as mean ± standard deviation. The two groups were compared statistically by unpaired double-tail *t*-test. Data between groups were compared using a one-way analysis of variance. It was deemed statistically significant when p < 0.05 was set.

## Results

### QL alleviates cardiomyocyte hypertrophy

**Fig. 1:**
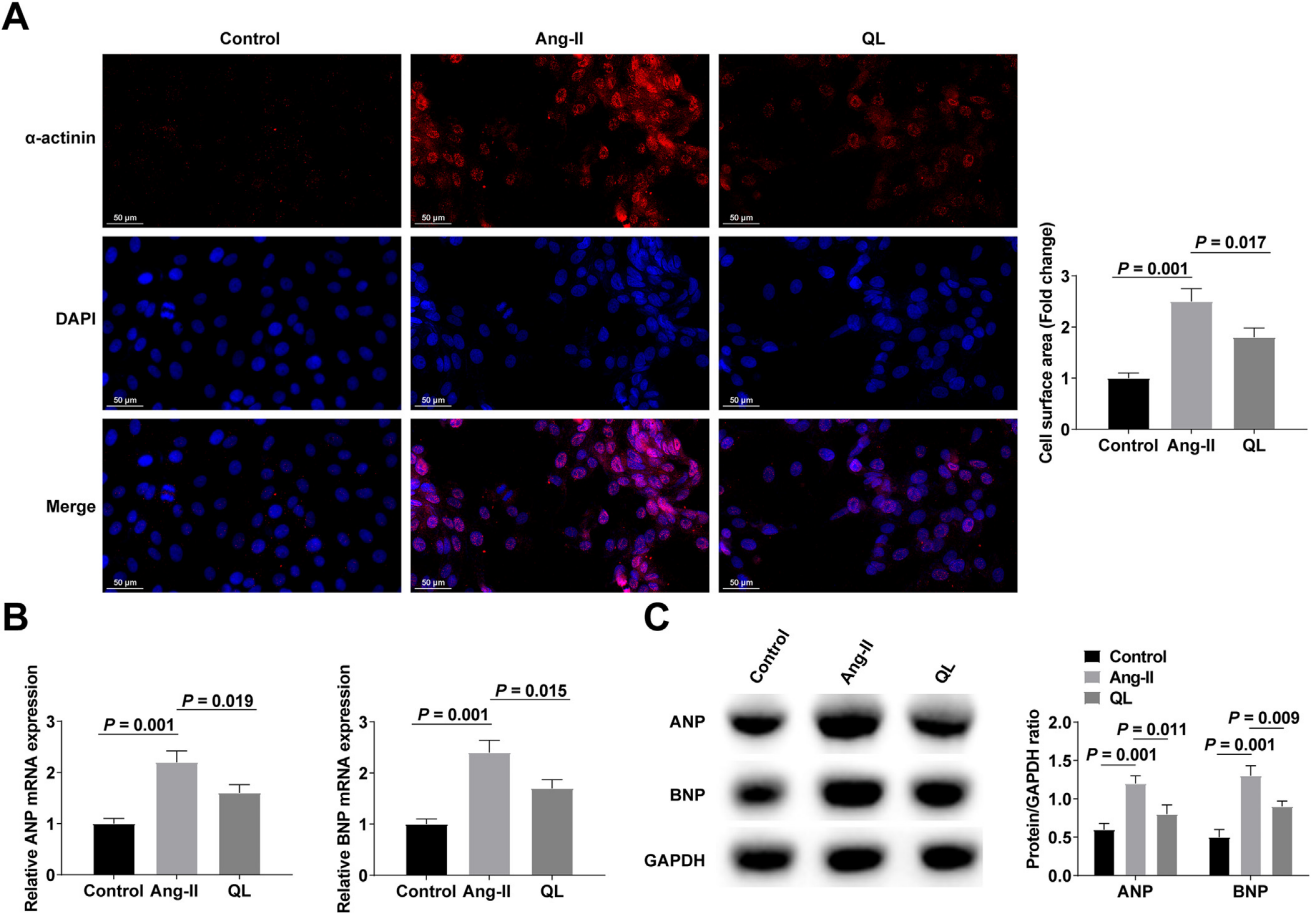
QL alleviates Ang-II-stimulated cardiomyocyte hypertrophy. (A) α-actinin immunofluorescence staining; (B‒C) RT-qPCR and Western blot measured ANP and BNP. Measurement data were shown in the form of mean ± standard deviation.

Cardiomyocyte hypertrophy was induced with Ang-II with or without QL preconditioning. Ang-II induced significant enlargement of cardiomyocytes by α-actinin-labeled cell surface measurements (Fig. 1A). Meanwhile, after Ang-II treatment, hypertrophic markers ANP and BNP continued to increase (Fig. 1B‒C). Interestingly, Ang-II-stimulated cardiomyocyte enlargement and increased expression of hypertrophic markers (ANP and BNP) were significantly reversed after QL treatment (Fig. 1A‒C), suggesting that QL had a protective effect on Ang-II-stimulated cardiac hypertrophy.

### Silencing miR-382-5p could further enhance the therapeutic effect of QL

**Fig. 2:**
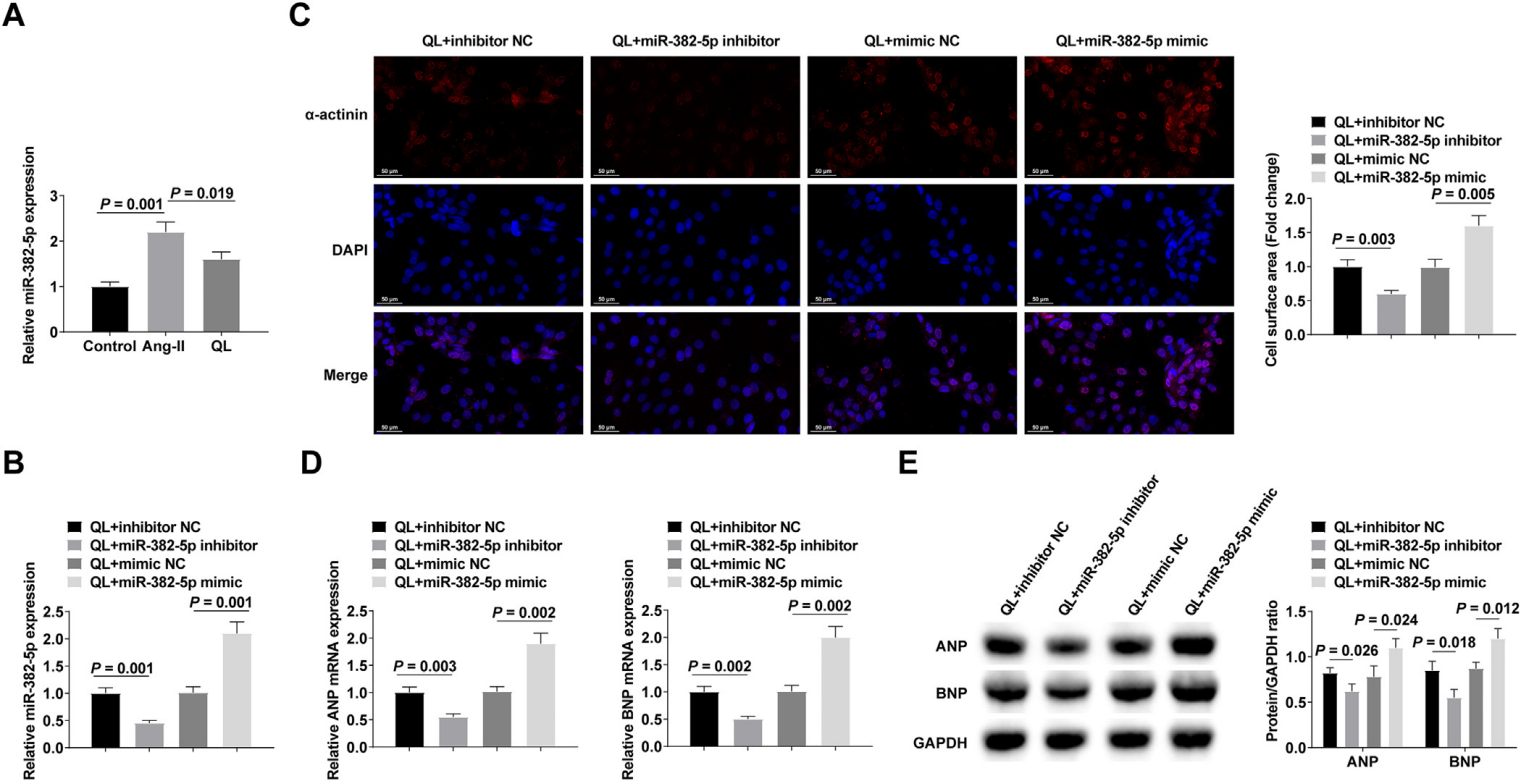
Silencing miR-382-5p can further enhance the therapeutic effect of QL. (A) RT-qPCR measured miR-382-5p; (B) RT-qPCR verified successful transfection; (C) α-actinin immunofluorescence staining; (D‒E) RT-qPCR and Western blot measured ANP and BNP. Measurement data were shown in the form of mean ± standard deviation.

miR-382-5p in NMVCs was analyzed by RT-qPCR, and the results indicated that miR-382-5p levels increased after Ang-II treatment, while QL treatment reversed this phenomenon (Fig. 2A). To probe the effect of miR-382-5p on cardiomyocyte hypertrophy, miR-382-5p inhibitor, inhibitor NC, miR-382-5p mimic, or mimic NC was transfected into QL-treated NMVCs. Successful transfection was verified by RT-qPCR (Fig. 2B). The therapeutic effect of QL could be further achieved by inhibiting miR-382-5p, whereas weakened by promoting miR-382-5p (Fig. 2C‒E).

### miR-382-5p negatively regulates ATF3 expression

**Fig. 3:**
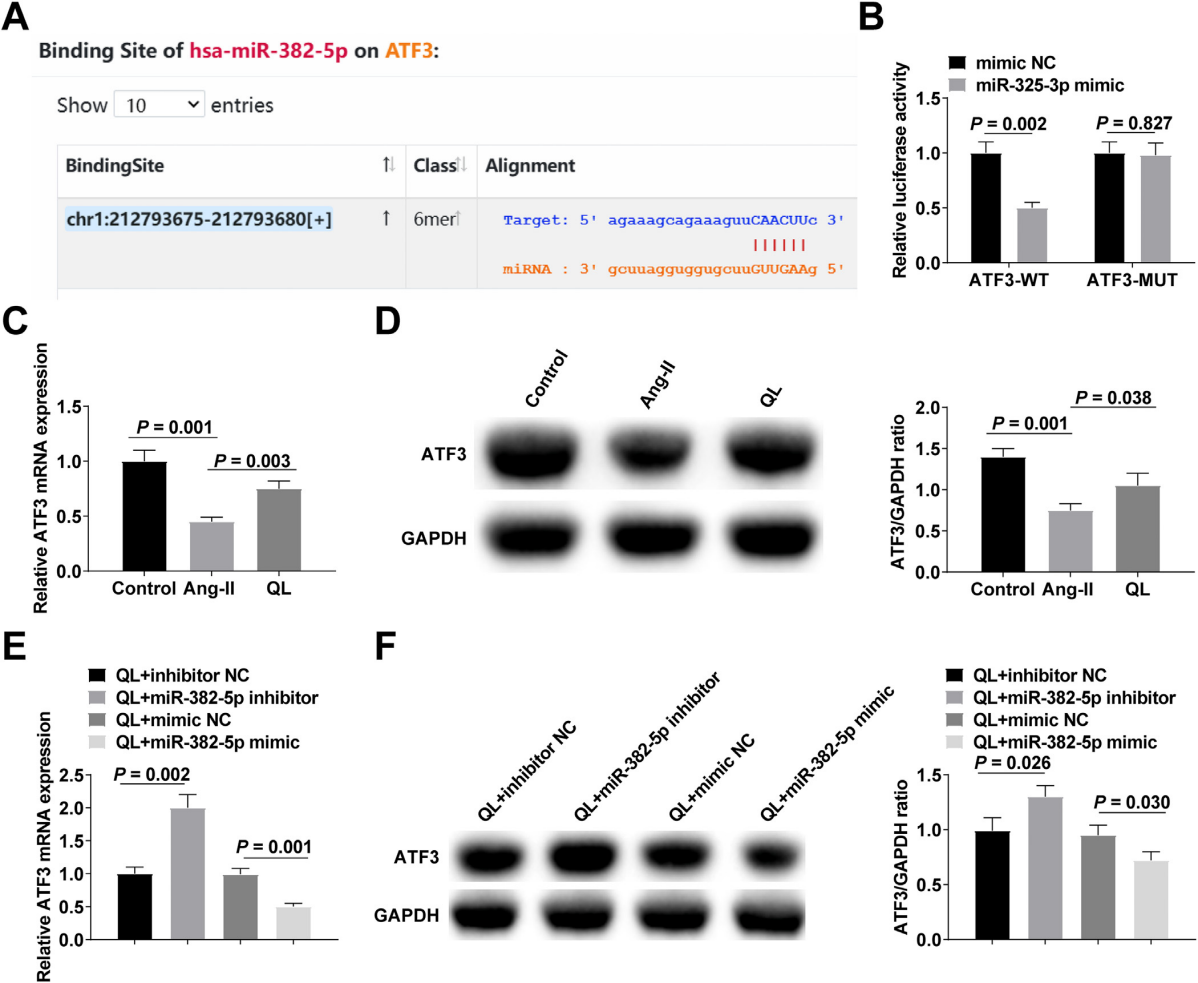
miR-382-5p negatively regulates ATF3 expression. (A) Bioinformatics website predicts targeted binding sites between miR-382-5p and ATF3; (B) Luciferase reporter gene experiment verified the targeting relationship between miR-382-5p and ATF3; (C‒F) RT-qPCR and Western blot measured ATF3 in NMVCs. Measurement data were shown in the form of mean ± standard deviation.

Then, the existence of targeted binding sites between miR-382-5p and ATF3 was predicted through the bioinformation website starBase (Fig. 3A). The findings from the dual luciferase experiment indicate a decrease in relative luciferase activity subsequent to the co-transfection of ATF3-WT and miR-382-5p mimic (Fig. 3B). ATF3 expression in NMVCs was quantified using both RT-qPCR and Western blot techniques. After Ang-II treatment, ATF3 expression decreased, while QL treatment promoted ATF3 expression (Fig. 3C‒D). ATF3 was increased after down-regulating miR-382-5p. After upregulation of miR-382-5p, ATF3 expression decreased (Fig. 3E‒F).

### ATF3 upregulation can reduce the hypertrophic effect of upregulation of miR-382-5p in NMVCs

**Fig. 4:**
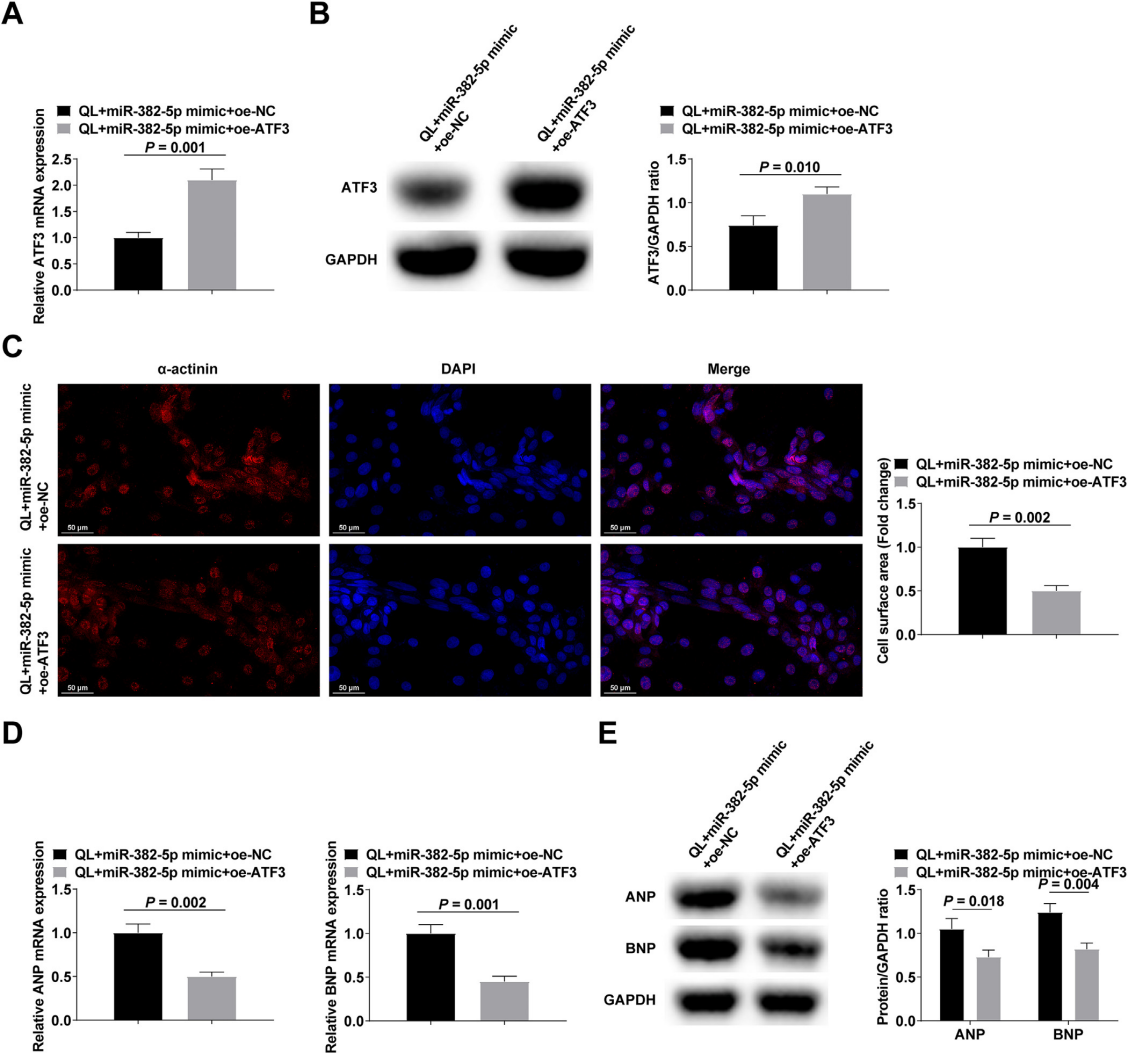
Upregulating ATF3 can reduce the pro-hypertrophic effect of upregulating miR-382-5p in NMVCs. (A‒B) RT-qPCR and Western blot verified transfection successfully; (C) α-actinin immunofluorescence staining; (D‒E) RT-qPCR and Western blot measured ANP and BNP. Measurement data were shown in the form of mean ± standard deviation.

NMVCs treated with QL were transfected with either miR-382-5p mimic + oe-ATF3 or miR-382-5p mimic + oe-NC. The confirmation of transfection efficacy was achieved by means of RT-qPCR and Western blot analysis (Fig. 4A‒B). The experimental results reported that elevating ATF3 could reduce the pro-hypertrophic impact of miR-382-5p upregulation in NMVCs (Fig. 4C‒E).

### QL alleviates cardiac hypertrophy and cardiac dysfunction in mice

**Fig. 5:**
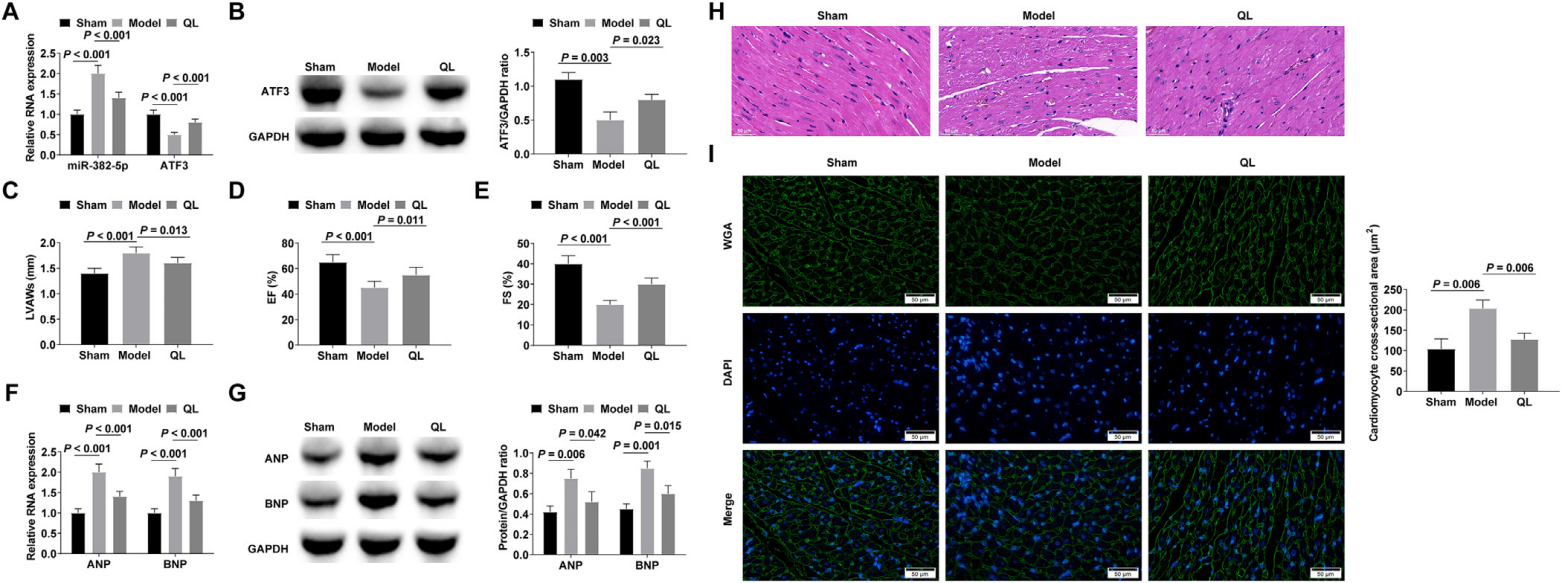
QL alleviates myocardial hypertrophy and cardiac dysfunction induced by Ang-II in mice. (A‒B) RT-qPCR or Western blot measured miR-382-5p and ATF3; (C‒E) Echocardiography detected cardiac function in mice; (F‒G) RT-qPCR and Western blot measured ANP and BNP; (H) HE staining observed the pathology of myocardial tissue in mice; (I) Cross-sectional area of cardiomyocytes observed by WGA staining. Measurement data were shown in the form of mean ± standard deviation.

To assess the *in vivo* therapeutic efficacy of QL, cardiac hypertrophy was induced in mice through the infusion of Ang-II for a duration of 3-weeks subsequent to a 4-week administration of QL. miR-382-5p was up-regulated and ATF3 was down-regulated in the hypertrophic myocardium, and QL treatment could inhibit miR-382-5p and promote ATF3 expressions (Fig. 5A‒B). Echocardiographic tests of cardiac function in mice showed that QL treatment effectively mitigated the Ang-II-induced augmentation of LVAWs and the decline in EF and FS (Fig. 5C‒E). RT-qPCR and Western blot confirmed that ANP and BNP increased in Ang-II-stimulated hypertrophic myocardium, while QL treatment decreased ANP and BNP levels (Fig. 5F‒G). After HE is staining, the myocardium induced by Ang-II showed typical hypertrophic changes, including collagen deposition in the myocardial extracellular matrix and inflammatory cell infiltration, while QL treatment significantly improved these pathological changes (Fig. 5H). Cardiomyocyte cross-sectional area was significantly increased in cardiac hypertrophied mice, whereas QL treatment reduced cardiomyocyte cross-sectional area (Fig. 5I).

### QL alleviates myocardial hypertrophy and cardiac dysfunction via the miR-382-5p/ATF3 axis

**Fig. 6:**
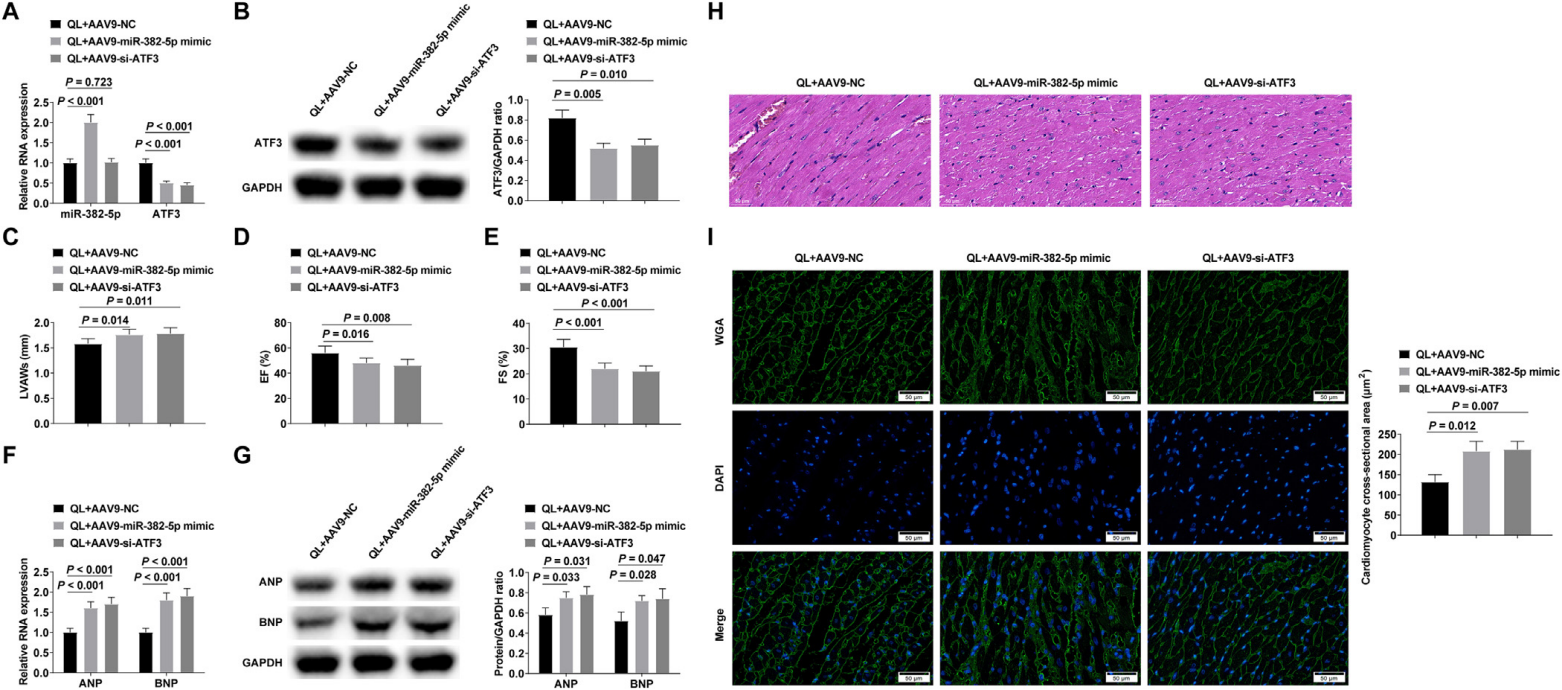
QL alleviates myocardial hypertrophy and cardiac dysfunction by regulating the miR-382-5p/ATF3 axis. (A‒B) RT-qPCR or Western blot verified successful adenovirus injection; (C‒E) Echocardiography detected cardiac function in mice; (F‒G) RT-qPCR and Western blot measured ANP and BNP; (H) HE staining observed the pathology of myocardial tissue in mice; (I) Cross-sectional area of cardiomyocytes observed by WGA staining. Measurement data were shown in the form of mean ± standard deviation.

Mice were injected with AAV9-NC, AAV9-miR-382-5p mimic or AAV9-si-ATF3 adenovirus through the tail vein after intragastric QL, and then injected with Ang-II to induce myocardial hypertrophy.. Successful adenovirus injection was verified by RT-qPCR and Western blot (Fig. 6A‒B). Experimental results proved that enhancing miR-382-5p or reducing ATF3 could reduce the therapeutic effect of QL (Fig. 6C‒I).

## Discussion

Heart failure and cardiac hypertrophy remain the leading causes of hospitalization and death among the elderly.^20^^,^^21^ Previous studies have established the efficacy of QL in diminishing NT-proBNP levels in heart failure patients, as well as mitigating cardiac remodeling and hypertrophy in diverse animal models.^22^^,^^23^ This present study provides novel evidence that QL alleviates myocardial hypertrophy and cardiac dysfunction through the miR-382-5p/ATF3 axis.

It is widely accepted that cardiac hypertrophy can be associated with different pathologies.^24^ QL has been shown to be effective in preventing cardiac dysfunction and remodeling in animal models of acute MI.^25^ QL-associated protection against cardiac remodeling and hypertrophy is thought to be due to several molecular mechanisms, such as inhibiting TNF-α/IL-10^5^ and angiotensin II type 1 receptor and activation of ErbB receptors,^9^ activating AMP-activated protein kinase/peroxisome proliferator-activated receptor-gamma coactivator-1alpha axis, NRG-1/Akt signaling,^8^^,^^22^ and PPAR-γ and mTOR signaling.^10^^,^^11^ Here, QL effectively alleviated Ang-II-stimulated cardiomyocyte hypertrophy, manifested by reduced cell volume and decreased ANP and BNP levels. Significantly, miR-382-5p expression was found to be elevated in hypertrophic cardiomyocytes stimulated by Ang-II, whereas its expression was inhibited by QL. Increasing evidence indicates that the aberrantly expressed miRNAs in pathologies could be used as potential biomarkers for disease diagnosis and prognosis prediction.^26^, ^27^, ^28^ For those diseases in which miRNA(s) was significantly upregulated, antagomiR(s) that could inhibit specific miRNA expression(s) in vivo may represent a novel therapeutic strategy.^29^ In line with these considerations, downregulation of miR-382-5p might be beneficial for the treatment of cardiac hypertrophy, which is at least in part responsible for the protective effect of QL. As the authors expected, miR-382-5p upregulation attenuated the anti-hypertrophic impact of QL in cardiomyocytes, whereas miR-382-5p downregulation yielded contrasting outcomes.

Translational silencing, translation inhibition, and/or degradation of target mRNA transcripts is usually accomplished by miRNA binding to the 3′-untranslated region.^30^, ^31^, ^32^ This study found the existence of targeted binding sites between miR-145-5p and ATF3. As a member of the cAMP response element-binding protein/ATF family,^33^^,^^34^ ATF3 acts as either a promoter or an inhibitor of transcription at the transcriptional level.^35^ Overexpressing ATF3 has significant anti-iron death and cardioprotective effects on hypoxic reoxygenation injury of cardiomyocytes.^36^ In this study, ATF3 was down-regulated in Ang-II-stimulated cardiomyocytes, and upregulating ATF3 could reduce the pro-hypertrophic effect of upregulation of miR-382-5p.

Finally, miR-382-5p was up-regulated in mice after Ang-II infusion, while ATF3 was down-regulated, and QL could inhibit miR-382-5p and promote ATF3 levels. Additionally, QL improved cardiac dysfunction induced by Ang-II, as measured by an increase in LVAWs and a decrease in EF and FS. Also, ANP and BNP increased in Ang-II-stimulated hypertrophic myocardium, while QL decreased ANP and BNP. QL can improve Ang-II-stimulated collagen deposition and inflammatory cell infiltration in myocardium extracellular matrix, and enhancing miR-382-5p or silencing ATF3 attenuated the anti-hypertrophy and cardioprotective effects of QL.

Limitations should be highlighted in this study. First of all, the study was only conducted in cells and animals, and the results cannot be extended to the clinic. QL, miR-382-5p, and ATF3 should be explored clinically for their roles in cardiac hypertrophy. Secondly, the downstream mechanism of ATF3 affecting cardiac hypertrophy is still unclear. Third, QL contains 11 different herbal ingredients, but further research is required to determine which compounds relieve cardiac hypertrophy.

## Conclusion

As demonstrated in this study, QL inhibits Ang-II-stimulated cardiomyocyte hypertrophy by miR-382-5p/ATF3 axis, but the mechanism of QL's protection against cardiac hypertrophy needs further study.

## Ethical statement

All animal experiments complied with the ARRIVE guidelines and performed in accordance with the National Institutes of Health Guide for the Care and Use of Laboratory Animals. The experiments were approved by the Institutional Animal Care and Use Committee of Zibo Hospital of Traditional Chinese Medicine (n° 20190626ZB).

## Consent to participate

Written informed consent was obtained from each subject.

## Consent for publishing

Written informed consent for publication was obtained from all participants.

## Authors’ contributions

Bao Yin designed the research study. Bao Yin and XiaoTong Jiang performed the research. XinFeng Chang provided help and advice. ChunHua Song analyzed the data. Bao Yin wrote the manuscript. ChunHua Song reviewed and edited the manuscript. All authors contributed to editorial changes in the manuscript. All authors read and approved the final manuscript.

## Funding

TCM Science and Technology Project of Shandong Province (2020Q082).

## Conflicts of interest

The authors declare no conflicts of interest.
